# Assessment Methods in Medical Ultrasound Education

**DOI:** 10.3389/fmed.2022.871957

**Published:** 2022-06-09

**Authors:** Elena Höhne, Florian Recker, Christoph Frank Dietrich, Valentin Sebastian Schäfer

**Affiliations:** ^1^Clinic of Internal Medicine III, Oncology, Hematology, Rheumatology and Clinical Immunology, University Hospital Bonn, Bonn, Germany; ^2^Department of Obstetrics and Prenatal Medicine, University Hospital Bonn, Bonn, Germany; ^3^Department of Internal Medicine, Clinics Beau-Site, Salem, and Permanence, Bern, Switzerland

**Keywords:** medical education, assessment, ultrasound, undergraduate education, practical skills

## Abstract

Medical schools are increasingly incorporating ultrasound into undergraduate medical education. The global integration of ultrasound into teaching curricula and physical examination necessitates a strict evaluation of the technology's benefit and the reporting of results. Course structures and assessment instruments vary and there are no national or worldwide standards yet. This systematic literature review aims to provide an up-to-date overview of the various formats for assessing ultrasound skills. The key questions were framed in the PICO format (Population, Intervention, Comparator, and Outcome). A review of literature using Embase, PubMed, Medline, Cochrane and Google Scholar was performed up to May 2021, while keywords were predetermined by the authors. Inclusion criteria were as follows: prospective as well as retrospective studies, observational or intervention studies, and studies outlining how medical students learn ultrasound. In this study, 101 articles from the literature search matched the inclusion criteria and were investigated. The most frequently used methods were objective structured clinical examinations (OSCE), multiple choice questions, and self-assessments *via* questionnaires while frequently more than one assessment method was applied. Determining which assessment method or combination is ideal to measure ultrasound competency remains a difficult task for the future, as does the development of an equitable education approach leading to reduced heterogeneity in curriculum design and students attaining equivalent skills.

## Introduction

Ultrasound examinations and obtained images are highly dependent on the physician's competence. Integration of ultrasound training offers opportunities to provide instruction in the use of novel educational and clinical practice tools and there is wide support for the incorporation into undergraduate medical education. However, despite growing interest in ultrasound education, course structure and implementation in undergraduate medical education programs differ between universities and countries without national standards and guidelines (1). A critical difficulty in ultrasound training is allocating time and funds for training programs in overburdened curricula. Early analyses demonstrated that in small cohorts, medical students were able to develop the psychomotor and interpretative skills required for effective focused ultrasound. For example, 1st year medical students were able to successfully use portable ultrasound after following six 90-min sessions covering abdominal, cardiovascular, genitourinary, and musculoskeletal applications (2). Recently, the European Federation of Medical and Biological Ultrasound Societies (EFSUMB) and the World Federation for Ultrasound in Medicine and Biology (WFUMB) have promoted undergraduate medical ultrasound education within European medical faculties and have developed measures to accomplish this objective (3, 4). The use of ultrasound in medical education depends on curricular requirements as well as the type of equipment available, the selected educational approach and faculty skill sets. These determine the type and quality of training delivered to students. Selecting an appropriate assessment method is crucial given the need to closely align learning objectives, instructional methods and exams. Reliable methods to assess physician's skills in performing ultrasound are critical for training and to prove the curriculum's quality (5). The global integration of ultrasound into medical education will make a regulated assessment and report of the results essential (6) in order to estimate and compare the efficacy of different attempts to organize ultrasound courses in medical education (7). There are various goals of assessment, including the optimization of learning and direct feedback in order to protect patients from insufficiently educated doctors (8) and to re-certify individuals, whose skills may have declined over time (9). However, a standardized method to evaluate ultrasound knowledge or of higher importance to assess the examination performance does not exist yet (9–11).

It has been proposed that the practical examination should include assessment of accurate machine settings, probe handling, image acquisition as well as documentation (12).

To assess various examination formats used for ultrasound in medical education, a systematic literature review of the MEDLINE, EMBASE, Cochrane, PubMed, and Google Scholar Databases was conducted to identify published literature on ultrasound assessment in undergraduate or graduate medical training.

## Materials and Methods

### Search Strategy

This systematic literature review was conducted according to the preferred reporting items for systematic reviews and meta-analyses (PRISMA) guidelines (13).

**Figure 1:**
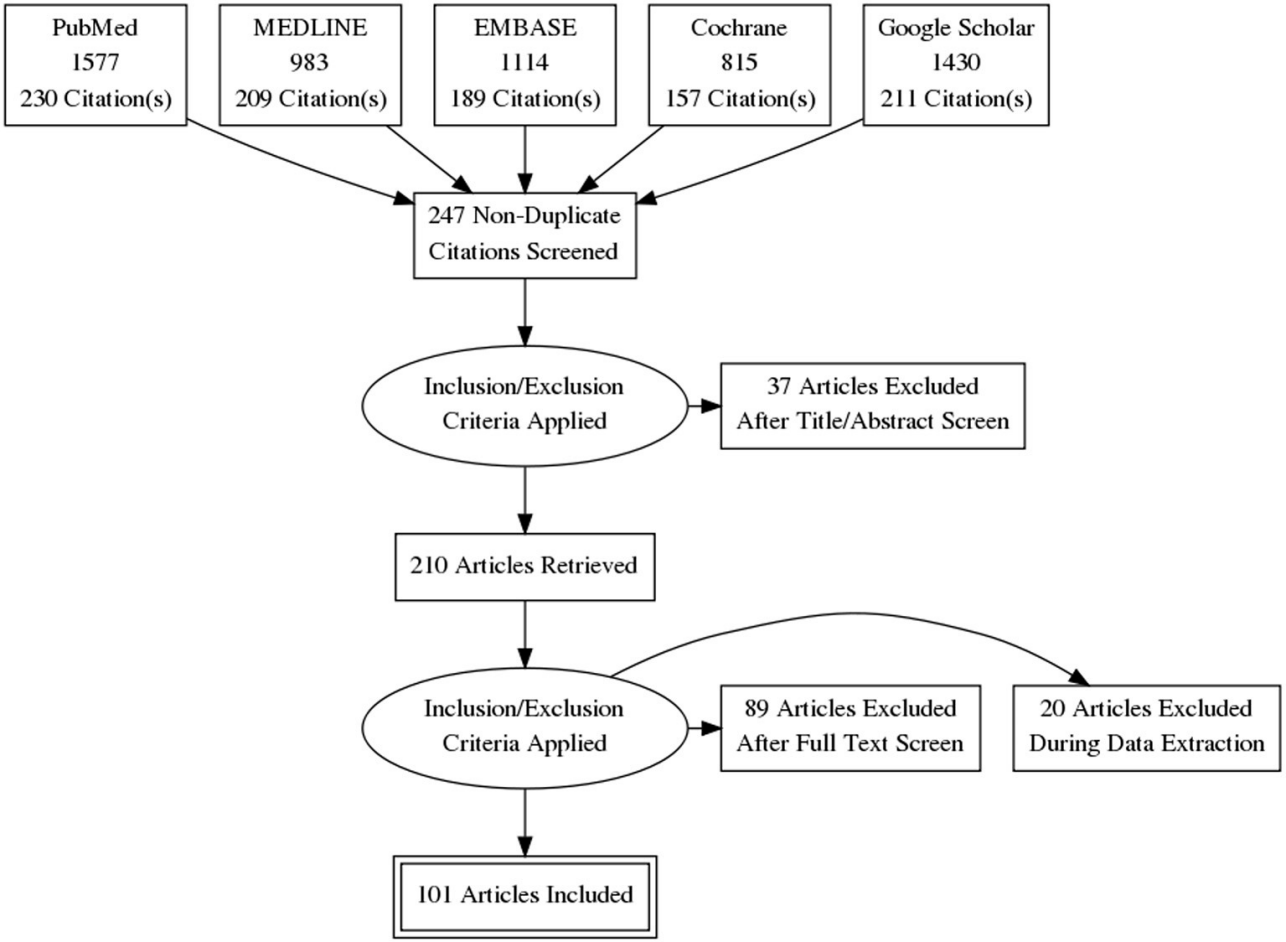
This systematic literature review was conducted according to the preferred reporting items for systematic reviews and meta-analyses (PRISMA) guidelines. The figure displays the review process, at the end 101 of 247 articles have been included.

Relevant medical databases, including PubMed, MEDLINE, EMBASE, Cochrane and Google Scholar were searched for publications related to the assessment of ultrasound skills up to May 2021, while keywords were predetermined by the authors (Figure 1). Titles and abstracts were analyzed for possible inclusion. In addition, reference lists of the identified articles were investigated for further potential inclusion. Agreement regarding potential relevance was reached by consensus and full text copies of relevant papers were obtained. The key questions of this systematic literature review were framed in the PICOS format (Population, Intervention, Comparator, Outcome, and Study design) as detailed in Table 1.

**Table 1:** Key questions of ultrasound methods and medical ultrasound education.

**Population**	Medical students, residents, physicians
**Intervention**	Ultrasound in education
**Comparator**	Different approaches to assess ultrasound skills
**Outcome**	Analysis of course structures and assessment instruments
**Study Design**	Prospective and retrospective studies, observational or interventional studies

### Inclusion Criteria

Articles meeting the following criteria were suitable for inclusion:

- Prospective as well as retrospective studies, observational or intervention studies, and studies outlining different assessment methods.
- The following keywords were combined: (ultrasound or sonography) and (education, medical) or (medical students) and (assessment) or (exam).

Exclusion criteria were:

- No data about the form of assessment or descriptive data only
- Duplicate articles within or between data bases
- Reviews
- Abstracts only
- Newsletters
- Conference presentations
- Expert opinions
- Editorials

The different assessment formats were structured in subclasses, which are the following: theoretical knowledge (written or online examination, multiple choice questions (MCQ) or essay questions), practical examination skills [e.g., US acquisition (US image rating), observed simulated clinical encounters (Objective Structured Clinical Examination (OSCEs), The Objective Structured Assessment of Ultrasound Skills (OSAUS), Direct Observation of Procedural Skills (DOPS))], and self-assessment (e.g., surveys regarding satisfaction and competence).

Data originated from full-text articles is presented in a structured table (Table 2) to illustrate the various assessment formats in ultrasound education and to give selected examples for the outlined evaluation methods.

**Table 2:** Example studies.

**References**	**Study design**	**Study site**	**Number of participants**	**Assessment**
Bernard et al. (14)	Prospective observational study	Loma Linda University School of Medicine	8	Post-instructional survey
Hammoudi et al. (15)	Prospective observational study	Faculty of Medicine Pierre et Marie Curie	348	Survey and open feedback
Hoyer et al. (16)	Single-center cross-sectional study	University of Arizona	55	Self-assessment by questionaire
Ivanusic et al. (17)	Prospective observational study	University of Melbourne	119	Survey and open feedback
Brown et al. (18)	Prospective observational study	University of Arizona	100	Survey and identification of US images
Keddis et al. (19)	Prospective observational study	Mayo Clinic Rochester, Minnesota	76	Pre- and post-survey
Rempell et al. (20)	Prospective observational study	Havard Medical School, Boston	176	Post-assessment survey
Swamy and Searle (21)	Prospective observational study	Durham University	215	Questionnaire
Teichgraber et al. (22)	Prospective observational study	Hannover Medical School	113	Questionnaire
Moscova et al. (23)	Prospective observational study	University of Sydney	901	Survey and open feedback
Dinh et al. (24)	Prospective observational study	Loma Linda University	163	Questionnaire, OSCE
Duanmu et al. (25)	Cross-sectional cohort study	Stanford University School of Medicine	29	OSCE
Hofer et al. (7)	Prospective observational study	H.-Heine University Düsseldorf	626	OSCE
Sisley et al. (5)	Prospective observational study	University of Arizona	82	OSCE
Knobe et al. (26)	Randomized controlled trial	RWTH Aachen University	151	OSCE, MCQ
Lozano-Lozano et al. (27)	Randomized controlled multicenter study	University of Granada	110	OSCE, MCQ, survey
Hofer et al. (28)	Longitudinal two cohort study	H.-Heine University Düsseldorf	2,485	OSCE
Gogalniceanu et al. (29)	Prospective observational study	Imperial College London	25	OSCE
Knobe et al. (30)	Randomized cross-over controlled trial	RWTH Aachen University	242	OSCE, MCQ
Bornemann (31)	Prospective observational study	University of South Carolina School of Medicine	17	OSCE, MCQ
Henwood et al. (32)	Prospective cohort study	Kigali, Rwanda	29	OSCE, image based assessment
Chuan et al. (33)	Prospective cohort study	Australian and New Zealand College of Anaesthestists	49	DOPS
Nilsson et al. (34)	Randomized controlled trial	University of Copenhagen	38	OSAUS
Royer et al. (35)	Prospective observational study	University of Colorado	32	Knowledge quiz, pre- and post-survey
Heinzow et al. (36)	Prospective observational study	University Hospital Münster	240	DOPS, pre- and post-survey
Hempel et al. (37)	Prospective observational study	Johann Wolfgang Goethe University Frankfurt	91	Questionaire to assess theoretical knowledge
Fox et al. (38)	Prospective controlled trial	University of California, Irvine School of Medicine	45	Image based test
Noble et al. (39)	Prospective cohort study	Massachusetts General Hospital	30	Image based test
Syperda et al. (40)	Prospective observational study	Lake Erie College of Osteopathic Medicine-Bradenton	5	Case based test
Madsen et al. (41)	Prospective observational study	University of Copenhagen	28	Assessment using virtual-reality ultrasound simulators
Yoo et al. (42)	Randomized controlled trial	University of Texas Southwestern Medical Center	28	Assessment using simulators and MCQ
Kobal et al. (43)	Prospective interventional study	University of California, Los Angeles	7	Comparison of findings between students and specialists
Mouratev et al. (44)	Prospective interventional study	University of South Carolina School of Medicine	14	Comparison of findings between students and specialists
Angtuaco et al. (45)	Prospective interventional study	University of Arkansas for Medical Sciences	24	Comparison of findings between students and specialists
Arger et al. (46)	Prospective observational study	University of Pennsylvania School of Medicine	33	Image rating
Mullen et al. (47)	Prospective observational study	California Northstate University College of Medicine	28	Real-time image rating
Tshibwabwa and Groves (48)	Prospective observational study	McMaster University Medical Center	490	Real-time image rating
Wittich et al. (49)	Prospective observational study	Mayo Medical School	42	Image rating
Fernández-Frackelton et al. (50)	Prospective observational study	Harbor-UCLA Medical Center	31	Image rating, pre- and post-theoretical test
Shapiroa et al. (51)	Prospective observational study	Mount Sinai School of Medicine	5	Image rating

*Data originated from full-text articles is presented in a table to illustrate the various assessment formats in ultrasound education and to give selected examples for the outlined evaluation methods*.

## Results

The results section that follows should provide a general understanding of the various assessment forms utilized in the evaluation of ultrasound.

### Self-Assessment

In many cases evaluation is based on self-assessment using surveys or questionnaires, sometimes in a pre-/post course design. Self-assessment can help to evaluate the student's thoughts and manners when learning and it can identify tactics that enhance better understanding and improvement of skills.

Further, students can rate their own competence, which might motivate them to improve their skills as they detect incongruity between present and wanted performance. According to one study, self-evaluation can help students improve their critical thinking skills (52) further it might encourage reflection on personal performance (53).

Creating surveys is time- and cost-effective and the evaluator does not essentially have to be a specialist. Since the evaluation is based on subjective data when using self-assessment, there is the threat of discrepancy between actual performance and answers given in the survey. Students might not rate their actual performance competence but the effort they put into the course (54).

There is no acquisition of genuine knowledge and competence in examination as no objective data is generated. Additionally, there is a risk of decreased validity by response bias which are prevalent in research involving participant's self-report. For example, the study results can be influenced by acquiescence bias, which belongs to response bias and describes that participants in a survey have the tendency to agree with the asked questions.

### Objective Structured Clinical Examination (OSCE)

The objective structured clinical examination (OSCE) was developed by Harden and colleagues in 1975 (55). The idea is rotating through multiple stations in a simulated clinical setting. Each of these stations challenges the student to solve a special task in a pre-specified time in order to test clinical skill performance. While the students carry out the examination they are observed by one or two assessors who rate the student's performance using checklists which have been developed in advance.

OSCE has been widely accepted as an objective form of assessing clinical competences (56) and is used in various specializations and clinical tasks.

It allows the assessment of scanning technique and image interpretation in real time and combines the evaluation of technical skills and theoretical knowledge. Further, the possibility for direct feedback on the student's performance is provided and it aims to prepare students for daily clinical practice. There are different assessors with every station and the students should rotate so that they should all have the same time and tasks to bring fairness.

Within an OSCE, different forms of assessment can be combined since e.g., at one station case- based US images could be diagnosed while the next task requires the students to examine a patient to obtain own images to observe the student's probe handling and scanning technique.

In general, the checklist for ultrasound can include various aspects, which have been defined prior to the assessment. Items that are often included are e.g., positioning of the patient as well as interaction with the patient, positioning/handling/orientation of the ultrasound probe, image adjustment, and interpretation. A different OSCE station is needed for each organ as the protocols are individually tailored.

Therefore, the large number of stations and the various protocols to test different organs as well as the required educated assessors may exceed the available resources (36). OSCEs require staff, equipment, clinical laboratories as well as long preparations. Furthermore, to avoid bias, two assessors each station would be preferable, resulting in even higher cost- and time investment. OSCEs might not display a realistic hospital setting (57).

As the time allotted to each station is predetermined, students may become stressed and be unable to complete the task to their own expectations owing to time constraints. However, limited time may compel a higher level of training motivation and motivate students to practice more in order to achieve satisfactory outcomes (7).

### Direct Observation of Procedural Skills (DOPS)

The concept of direct observation of procedural skills (DOPS) was developed by the Royal Medical College of England. The assessor observes the student during the clinical procedure on a real patient and gives feedback afterwards, it is a workplace-based assessment method.

Further, DOPS combines learning, supervision, rating and feedback (58) and can be both formative and summative (59).

Designing specific protocols for grading facilitates detailed feedback and fairness since the assessor has the same base to rate the different students.

The use of workplace-based assessment is useful since it determines not only the students' learning achievements but also their attempt to assume professional responsibilities (60). Scanning technique and image interpretation can be assessed in real time and therefore theoretical knowledge as well as examination technique can be rated. DOPS have been used for ultrasound assessment and seems to be a reliable and valid method (36) and requires less assessment stations and resources than an OSCE format. However, an educated assessor is necessary for the evaluation of the student and it would be even better to have two independent raters. Further, in comparison to OSCE, DOPS is not widely established in ultrasound assessment and there might be more studies required to show its efficacy and validity.

### Objective Structured Assessment of Ultrasound Skills (OSAUS)

The Objective Structured Assessment of Ultrasound Skills (OSAUS) was developed as an approach to achieve international consensus across various specialities on an evaluation tool for ultrasound education (9). Based on a delphi-consensus seven key points have been identified and included in the protocol.

These key points are: (1) Indication for the examination, (2) Applied knowledge of ultrasound equipment, (3) Image optimization, (4) Systematic examination, (5) Interpretation of images, (6) Documentation of examination, and (7) Medical decision making.

OSAUS can be used to assess US competence in different clinical settings and disciplines. It is a time-effective method as the protocol should be used universally there is no need of developing a new protocol for various specializations/organs. However, OSAUS is measuring general aspects and is not procedure specific.

The student is asked, to state the indication for the examination, as well as how the examination could help in further decision making and treatment. Therefore, the student learns the importance of ultrasound and the practical applications of this imaging method.

Further studies are required to examine the value of the protocol to assess ultrasound competence in different fields and clinical settings.

### Multiple Choice and Written Questions

Examinations in form of multiple choice (MCQ) or written questions are often used additionally to a direct observation of scanning technique (26, 27, 61).

Using MCQ is objective and the questions could be included in any existing examination.

Further, no educated assessor is required for the real-time assessment of skills.

Due to the absence of an ultrasound examination, evaluating scanning method and image optimization is not feasible. Whereas, ultrasound is a technical skill, the assessment with MCQ rather checks theoretical knowledge.

### Images and Case-Based Questions

Including pictures and case-based assessments are more opportunities for the assessment of ultrasound knowledge. Here, students have to detect pathologic findings in US images and have to connect them to clinical cases and further clinical applications.

Short duration and case based presentations can increase the knowledge maintained after 2 weeks in learning (37).

Case based learning (CBL) is a teaching method which finds application in multiple medical fields using case vignettes to convey relevance and to connect theory to practice (62).

It is objective and does not require any special educated assessor. Just like MCQ, case-based questions can be incorporated into an already existing exam.

Since it is a cost- and time-effective tool it can be well-used when resources are limited.

However, probe handling, scanning technique and image adjustment cannot be evaluated with this method of assessment and would be necessary to further improve the students' competence.

### Skills Assessment on Simulators

Several studies have shown that simulation-based ultrasound training can lead to better clinical performance not only regarding diagnostic accuracy, but further students seem to need less supervision (63). Ultrasound simulators are important for training in anesthesia and gynecology and can be used for the assessment of competence (41). They are used especially for rather advanced exercises e.g., for the incorporation of central venous catheters or practicing regional anesthesia (64) as they provide the possibility to practice complex tasks prior to the performance with patients. Simulators can offer standardized and valid measurement of skills that can be compared not only nationally but globally (41). The clinical setting is missing and the interaction with the patient cannot be evaluated. On the other hand, the assessment in a clinical setting requires expenditure not every university can afford.

When the number of simulators is limited, the assessment can be affected if the students memorized the correct position. Since anatomy differs, the learning effect when scanning patients might be different.

### Comparison of Findings Between Students and Specialists

In this assessment format students as well as experts were asked to perform the same examination and results were compared afterwards.

One study trained students to measure the liver size using ultrasound. Experienced physicians were asked to measure liver span with standard examination methods. Afterwards the results were compared to the student's findings to evaluate if the course was effective (44). Another study compared the precision of cardiovascular diagnoses by medical students using a mobile ultrasound device with the findings of cardiologists which were using standard physical examinations (43). This assessment form provides a clinical setting and students get to perform examinations with real patients. Theoretical knowledge cannot be assessed neither can scanning technique or probe handling since only the resulting image is rated. In this case the students do not get direct feedback on their performance.

### Rating of Images

In some studies US images of the students were rated by predetermined criteria. For example images from the pretraining scanning examination and the images from the post-training scanning examination were stored and then compared for improvement (46). In other studies the image quality was live-evaluated and students got a different score, depending on their ability to visualize the organ (47, 48).

There is no global accepted rating system of US images yet, even though the need for a standardized method to evaluate the quality of an US image is well-documented. The B-QUIET method for example represents such an approach to quantify the sonographer component of ultrasound images (65) (Table 3).

**Table 3:** Ultrasound assessments in medical education.

**Form of assessment**	**Positive aspects**	**Negative aspects**
Self-assessment or surveys regarding satisfaction	• Easy to create and evaluate • No need of a specialist as assessor • Cost effective	• Only subjective elements are measured, no objective view • No direct feedback to the student • No information about actual knowledge or practical skills • Bias possible, depending on the question structure
OSCE	• Assessment of both scanning technique and image interpretation • Combines evaluation of technical skills and knowledge in real time • Direct feedback to the students • Can connect different assessment forms: case based questions e.g., could be incorporated • Widely used, not only for US but for the assessment of multiple practical skills	• Requires different stations and protocols if different organs/situations shall be presented • Requires assessor who is educated in US and assessment • Better even to have different assessors to prevent bias, therefore high cost- and time expenditure
DOPS	• Assessing skills in a workplace setting • Formative and summative, observing knowledge and skills • Direct feedback	• Requires assessor to rate student, better even more than one • Not widely established yet, might need more studies showing efficiency/validity
OSAUS	• Objective measurement tool • Protocol is applicable for different specializations and clinical situations • Not only focused on direct performance at scanning, further checks if the student has the needed knowledge to evaluate if the US examination is necessary and how it could help in the further treatment of the patient • Approach for global rating system –> delphi consensus • Rating system which has been developed for US only	• Since it should be applicaple for different specializations it is more general than e.g., osce protocols since not every special finding for the different organs are named • Experienced assessor needed, not widely established yet
multiple choice and written questions	• Objective • Can be incorporated into another exam (e.g., internal medicine) • No special educated assessor necessary	• If used alone no direct evaluation of scanning technique • Knowing what is shown on an US image or how a disease would show does not mean that the student is capable to obtain the image and detect the pathology • US is a technical skill while MCQ rather checks theoretical knowledge
Pictures and case based questions	• Objective • Has been shown to be a good learning strategy • No assessor necessary • Can be incorporated into another exam	• No information about how the students' competence in an examination would be • No information about students' probe handling/ image acquisition
Skill assessment on simulators	• No accidental findings which could be detected when scanning other students • Good training prior to examine a real patient, especially for rather advanced tasks	• No clinical setting • Better learning effect while scanning real humans • Might know the simulator from training and memorizes locations
Comparison of findings between students and specialists	• Examination of real patients • Clinical setting • Objective • No theoretical approach but students had to obtain images	• No direct feedback • No check on scanning technique, only results are compared
Rating of images	• Direct outcome is evaluated • Practical skills are assessed • Objective	• Examination itself is not evaluated, therefore no direct feedback on scanning technique • Theoretical knowledge is not evaluated and no globally accepted image rating system is existing yet

*This overview depicts the various forms of evaluation, as well as the benefits and drawbacks of the various approaches in medical ultrasound teaching*.

## Discussion And Conclusion

Since assessment is a crucial part of medical education and plays a major role in the concept of constructive alignment, it should always be considered in curriculum development. Especially for practical skills such as ultrasound it plays a vital role and it is essential to ensure efficacious use of this specific technology (66). As ultrasound is a hands-on skill, the examination should ideally not only ask for theoretical knowledge, in addition it may be necessary to assess the resulting ultrasound images or the scanning technique. Furthermore, when testing, one should make sure that not only subjective outcomes are assessed to ensure that the study results can be used to compare different teaching methods in order to find the best educational approach. By using a combination of different assessment methods, some of the limitations that every examination format has can be compensated, learning objectives can be elaborated and inadequate performance can be detected (67). As seen by the numerous assessment forms presented, there is currently no internationally acknowledged assessment tool. Future studies should analyze and develop consensus on when and how ultrasound can be utilized effectively, as well as how ultrasound assessment should be incorporated into medical school education. Moreover, a general approach of an equal education in different universities and countries leading to less variability in the curriculum design is needed. There have already been attempts to develop a single general assessment tool that could be used to evaluate ultrasound skills across diverse specializations and contexts (9, 65). The decision which assessment method or which combination is best to measure ultrasound competency remains a challenging task for future trials. Besides, standards have to be defined as well as the frequency at which students should be tested.

## Data Availability Statement

The original contributions presented in the study are included in the article/supplementary material, further inquiries can be directed to the corresponding author.

## Author Contributions

Material preparation, data collection, analysis were performed, and the first draft of the manuscript was written by EH, FR, and VS. CD and VS helped by manuscript editing. All authors contributed to the study conception and design, commented on previous versions of the manuscript, read, and approved the final manuscript.

## Conflict of Interest

The authors declare that the research was conducted in the absence of any commercial or financial relationships that could be construed as a potential conflict of interest.

## Publisher's Note

All claims expressed in this article are solely those of the authors and do not necessarily represent those of their affiliated organizations, or those of the publisher, the editors and the reviewers. Any product that may be evaluated in this article, or claim that may be made by its manufacturer, is not guaranteed or endorsed by the publisher.
